# The Potential of Virtual Reality-Based Multisensory Interventions in Enhancing Cognitive Function in Mild Cognitive Impairment: A Systematic Review

**DOI:** 10.3390/jcm14155475

**Published:** 2025-08-04

**Authors:** Maryam Mehrinejad Khotbehsara, Jeffrey Soar, Sachithra Lokuge, Elham Mehrinejad Khotbehsara, Wing Keung Ip

**Affiliations:** 1 School of Business, University of Southern Queensland, Ipswich, QLD 4300, Australia; maryam.mehrinejadkhotbehsara@unisq.edu.au (M.M.K.); jeffrey.soar@unisq.edu.au (J.S.); sachithra.lokuge@unisq.edu.au (S.L.); 2 School of Engineering, University of Southern Queensland, Toowoomba, QLD 4350, Australia; 3 School of Culture Community and Health, University of Bedfordshire, Luton LU1 3JU, UK; benson.ip@beds.ac.uk

**Keywords:** mild cognitive impairment, cognitive function, executive function, virtual reality, functional ability

## Abstract

**Background:** This systematic review investigates the role of virtual reality (VR)-based multisensory cognitive training in cognitive function, executive function and wayfinding ability among people diagnosed with mild cognitive impairment (MCI) and Alzheimer’s disease (AD). **Methods:** The review was carried out using PRISMA guidelines. PubMed, Scopus, Embase, and Google Scholar were searched up from inception to February 2025 using terms related to MCI, AD, VR, and cognitive functions. Studies were included if they involved participants with MCI or early AD, used VR-based training, collected baseline data, and reported cognitive outcomes. **Results:** Nine studies with MCI were included, but no eligible studies focused on AD. Seven out of nine eligible studies in MCI reported significant improvements in global cognitive function (MoCA, CERAD-K, MMSE). Some studies showed improvements in executive function (EXIT-25, TMT-A/B, and SCWT), while others found no significant differences. One study reported improved depression/mental status (GDS, MOSES, QoL-AD). Just one study reported improvement in functional ability (IADL). One study reported enhanced cognition and reduced discomfort (SSQ). VR programs were generally well-tolerated, with no significant adverse events reported. **Conclusions:** VR shows promise for improving cognitive function in MCI. VR also showed potential benefits in executive function and psychological outcomes like depression and quality of life, though consistency varied.

## 1. Introduction

MCI and early-stage AD mark crucial phases in the development of neurodegenerative conditions, characterized by noticeable declines in cognitive abilities like memory, executive functioning, and spatial navigation. MCI impacts about 15 to 20 percent of individuals over 65, with 10 to 15 percent progressing to dementia each year [1,2]. AD is the most common form of dementia, contributing to 60–80% of all diagnosed cases and affects over 55 million individuals worldwide [3,4]. Among the various cognitive challenges linked to MCI and early AD, wayfinding is particularly impacted, with studies showing that up to 80% of individuals with MCI and AD in early-stage experience significant difficulties in spatial navigation [5,6]. These deficits not only compromise the autonomy and well-being of those affected but also contribute to heightened risks of disorientation, falls, and social isolation, placing a substantial burden on caregivers and healthcare systems [7,8]. VR has recently gained recognition as a powerful and innovative tool in cognitive rehabilitation and neuropsychological research, providing a unique platform to create virtual environments for real-world navigation tasks, like city streets or buildings, for individuals with cognitive impairments like MCI and early AD [1,9]. For individuals with MCI and early AD, VR-based interventions significantly improve wayfinding skills, spatial cognition, and navigation abilities through repetitive, adaptive, and task-specific exercises [10]. Clinicians can also obtain precise measurements of performance metrics, such as reaction time, path accuracy, and error rates, to track progress and tailor interventions to patients’ needs in both clinical and home-based settings [11,12]. The theoretical concept of VR follows neuroplasticity posits that brain retains the capacity of reorganization of neural connections through activate hippocampal regions, even in the presence of neurodegenerative diseases, so VR by providing multisensory simulation can improve cognitive functions by enriching environments and induce some delays in disease progression [5,9,10]. Studies demonstrate that VR-based training with scalability advantages provide improvement in cognitive performance by integrating visual, auditory, and sometimes haptic cues in AD participants [9,11]. Nonpharmacological strategies such as regular physical activity, a healthy diet like the Mediterranean diet, and adequate sleep are closely associated with improved cognitive function and a reduced risk of mild cognitive impairment and dementia. In contrast, insufficient sleep is recognized as a significant risk factor for the development of AD [13,14,15,16,17]. Although virtual reality has shown promise in enhancing cognitive function among older adults, its effectiveness tends to be less significant compared to the well-established benefits of lifestyle habits like physical activity, healthy eating, and adequate sleep [13,18,19]. While some studies report significant improvements in navigation abilities following VR-based training, others highlight the need for larger, randomized controlled trials (RCTs) to establish more robust evidence in MCI [12,20,21,22]. Although research in this area continues to expand, the use of VR to improve wayfinding skills in disorders associated with neurodegeneration such as MCI and AD, there remains a lack of consensus due to variations in study designs, VR protocols, and outcome measures [22]. These inconsistencies underscore the importance of conducting a systematic synthesis of the evidence to identify trends, address methodological limitations, and offer a better understanding of how effective VR strategies are. Previous reviews have mainly focused on feasibility [23], general cognitive outcomes, or RCTs, often overlooking smaller or single-arm pre-post studies [24] that may still yield important insights. Considering this gap, this review aims to investigate the impact of VR-based multisensory cognitive training on cognitive function, executive function, and wayfinding ability in individuals with MCI and AD, based on pre- and post-intervention assessments from either single-group or randomized study designs with the goal of informing future research and clinical practice. Accordingly, this study aims to address the following research question: How does VR-based multisensory cognitive training influence cognitive, executive, and wayfinding abilities in individuals with MCI and AD, based on pre- and post-intervention outcomes?

## 2. Materials and Methods

### 2.1. Search Strategy

The protocol for this systematic review was registered with the International Prospective Register of Systematic Reviews (PROSPERO) prior to the commencement of the study, under the registration number CRD420250651300, to ensure transparency and avoid duplication. This study, conducted in line with PRISMA (Preferred Reporting Items for Systematic Reviews and Meta-Analyses) guidelines [25], systematically reviews published studies on the influence of VR-driven multisensory intervention on cognitive abilities in individuals with MCI and early AD. An extensive search was carried out among several online databases, including PubMed, Scopus, Embase, and finally checking Google Scholar to not miss any studies from the inception of the respective database up to February 2025, to identify studies in English. The following MeSH terms were used to search the databases: Mild Cognitive Impairment, Alzheimer’s disease, Wayfinding Skills, Spatial Memory, Virtual Reality, Navigation Accuracy, Memory Improvement, Orientation, Cognitive Functioning, and Executive Function. Duplicates, reviews, case reports, case series, conference and congress abstract, editorial correspondence, comments and observational studies were excluded in the beginning. The screening process was carried out independently by two reviewers, who first assessed the titles and abstracts. Studies that appeared to meet the initial criteria were then examined in full. Articles were organized based on their MCI and AD according to our PICO criteria. The following criteria were used to determine study eligibility: P (population): patient with MCI or AD, I (intervention): Treatment with VR-based training, C (comparison): patients before taking intervention, O (outcome): Wayfinding Skills, Navigation Accuracy, Spatial Memory, Memory Improvement, Orientation, Cognitive Functioning, Executive Function.

### 2.2. Eligibility Criteria

Inclusion: (1) Publications available in the English language; (2) involved participants diagnosed with MCI or early-stage AD based on standardized diagnostic criteria, implemented via VR-based interventions incorporating at least one multisensory component (e.g., visual, auditory, or haptic stimuli); (3) targeted cognitive function, including domains such as memory, executive function, spatial navigation, or wayfinding; (4) reported pre- and post-intervention assessments of cognitive or functional outcomes using validated instruments; (5) provided sufficient methodological detail, including intervention protocol and outcome measures, to allow for data extraction and quality appraisal.

Exclusion: (1) Advanced AD or severe dementia; (2) significant sensory or motor impairments preventing VR use; (3) other neurological or psychiatric conditions unrelated to MCI/AD; (4) history of severe motion sickness or inability to tolerate VR; (5) healthy individuals or those with non-MCI/AD cognitive decline, (6) non-VR intervention or lack of multisensory elements; (7) non-specific focus interventions; (8) inaccessible formats of interventions; (9) non-specific control group or control group with unrelated interventions.

### 2.3. Extracting Data

M.M. and E.M., the two reviewers, gathered information on the study’s characteristics. The general information extracted for all outcomes included: study characteristics (author, country and year), demographic information of study population (sex and age), sample size, follow up duration, type of outcomes, and results. Quality of Life, Self-Reported Measures, Independence, Emotional and Psychological Impact, Confidence in Wayfinding, Mood or Anxiety, Feasibility, Safety Issues, Engagement and Adherence were considered as second outcomes.

### 2.4. Study Selection

**Figure 1:**
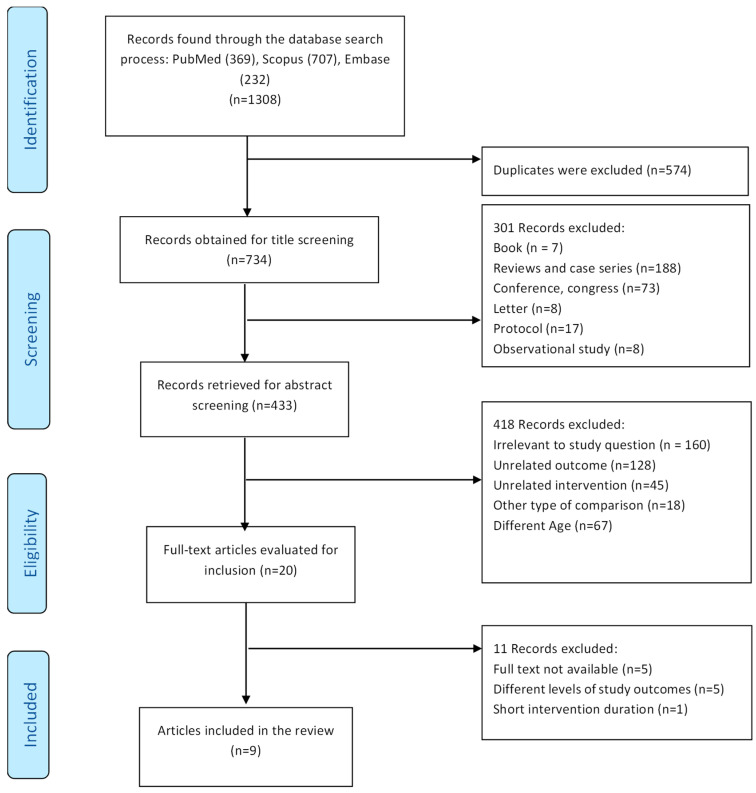
Flowchart based on PRISMA guidelines illustrating study identification and screening study selection.

The initial search yielded 1308 records. Specifically, 369 records were found in PubMed, 707 in Scopus, and 232 in Embase. Of these, 566 studies were excluded due to duplication. After reviewing the titles and abstracts, an additional 722 reports were excluded due to mismatches in outcomes and interventions, age, and study types. Out of the remaining 20 studies, 5 were excluded because full text access was limited, and another 5 studies involved participants whose cognitive decline appeared more advanced than what is typically seen in MCI, which did not meet our criteria. One study was removed because the intervention duration was too brief to meet the minimum required threshold for assessing its effectiveness. As a result, we included 9 studies that met the eligibility criteria and addressed MCI in our systematic review. These articles focused on studies involving older adults aged 65 and older with MCI who had undergone VR interventions. Although our search strategy and eligibility criteria were designed to include both MCI and early-stage AD populations, no studies meeting all criteria were identified for the AD group. Therefore, only studies involving MCI are included in this systematic review and data synthesis. Figure 1 shows the process of database searching, screening, and the selection of studies in PRISMA guidelines that met the eligibility criteria.

### 2.5. Assessment of Risk of Bias

Consistent with the non-randomized before-and-after design of the included studies, risk of bias was assessed using the National Heart, Lung, and Blood Institute (NHLBI) study quality evaluation tool for pre–post studies [26]. This tool includes 12 questions designed to evaluate the study’s aim, sampling methods and the size of sample, description of intervention and outcome, blinding, follow-up, and statistical methods. The possible answers to these questions include yes, no, not reported (NR), cannot be determined (CD) and not applicable (NA). The overall score is categorized as Good (score of more than 8), Fair (score of 5 to 8), or Poor (score less than 5). The same reviewers conducted the risk of bias assessment for the included studies and verified their findings. The evaluation of study quality is summarized in Table 1.

The questions address the following aspects: (1) the clarity of the study objective, (2) specification of eligibility criteria, (3) representativeness of participants, (4) inclusion of all eligible individuals, (5) adequacy of sample size, (6) description of the intervention, (7) appropriateness of outcome measures, (8) use of blinded assessors, (9) follow-up rate, (10) appropriateness of statistical analysis, (11) use of multiple outcome measures, and (12) availability of individual-level data to assess group effects.

## 3. Results

### 3.1. Description of Selected Studies

The studies chosen for review include two articles from Korea, two from Taiwan, and one each from China, Japan, Canada, Estonia, and Ecuador. Most studies focused on VR [20,29,30,31]; however, some also explored variations of these approaches, such as Immersive VR-based cognitive training (VRCT) [12,21,27], Virtual Supermarket (VSEE) [22], and VR-based cognitive–motor rehabilitation (VRCMR) [28]. Considering the pre-post study design for inclusion criteria, there were no eligible studies for AD patients. A summary of the key features of the included studies is shown in Table 2.

### 3.2. Cognitive Function

Most studies (7 out of 9) evaluated cognitive function. Five of these studies utilized the Montreal Cognitive Assessment (MoCA) score [20,22,27,28,29], while the remaining studies employed the Consortium to Establish a Registry for Alzheimer’s Disease-Korean Version (CERAD-K) and the Mini-Mental State Examination (MMSE) tools [12,31]. The results of all seven studies assessing cognitive function indicated significant improvements following the VR intervention [12,20,22,27,28,29,31].

### 3.3. Executive Function

Five articles evaluated executive function using various assessment tools, including the EXIT-25 [27], TMT-A/B [21,28,29,31], SCWT [21], working memory tests, and measures of cognitive flexibility [29]. In a study that evaluated executive function with the EXIT-25, scores on this tool decreased following the VR intervention, suggesting an improvement in executive function [27]. Notable improvements in cognitive function were reported by two additional studies, as assessed using the TMT-A/B and SCWT tools [21,28]. Conversely, two studies reported no significant changes in executive function following the VR intervention [29,31].

### 3.4. Spatial Navigation

Although no studies included in this review specifically measured wayfinding or spatial navigation, a few studies involved tasks that are closely related to these abilities. One study [21] showed improvements in executive functioning and dual-task gait performance, using the TMT-B, which taps into skills like cognitive flexibility and sequencing, both of which are essential when navigating complex environments. Another study [22] used a VSEE simulation, which replicates real-world spatial challenges and likely engaged participants’ orientation and spatial memory. In addition, findings from VRCMR in a virtual setting revealed notable improvements in planning and working memory, based on tests like the TMT and digit span tasks [28]. These improvements suggest that while wayfinding wasn’t directly assessed, the VR-based interventions may still have enhanced some of the underlying cognitive abilities needed for successful navigation, particularly in people with MCI.

### 3.5. Functional Ability

Functional ability was measured using the IADL scale in two studies. This tool evaluates areas such as communication, self-care, financial skills, physical mobility, health-related tasks, and everyday activities. In one of these studies, significant improvement in IADL was observed after the VR intervention [27]; however, in the other study, no statistically significant difference was found following the intervention [20].

### 3.6. Psychological Outcomes

Three studies evaluated psychological outcomes, specifically depression and quality of life. The Geriatric Depression Scale (GDS) [12,20] and the Multidimensional Observation Scale for Elderly Subjects (MOSES) [31] were used to evaluate depressive symptoms. The Korean adaptation of the Quality of Life–Alzheimer’s Disease (QoL-AD) scale was used to assess quality of life [12]. In one study, improvements in patients’ depression and mental status were observed [20]; however, the other two studies found that the intervention did not affect depression or quality of life [12,31].

### 3.7. Safety Evaluation Scale

These studies utilize a simulation tool known as SSQ to evaluate safety and potential side effects experienced in virtual environments. In one study, the VR program was found to enhance cognition and reduce discomfort in all SSQ items among the MCI group [12]. However, another study reported no significant improvements [22].

### 3.8. Feasibility of VR Programs

Several studies have investigated the feasibility of VR. All studies indicated the absence of significant negative outcomes. Most individuals found the VR experience engaging and observed benefits related to both their physical and cognitive functioning [30].

### 3.9. Results of Quality Assessment of Included Studies

To assess the quality of the nine included studies, we utilized a standardized tool designed for evaluating before–after (pre–post) studies lacking a control group. Among these, four studies (44.4%) were classified as high quality, while the remaining five (55.6%) were deemed to have moderate quality. All studies clearly articulated their research aims and participant inclusion criteria. In eight studies (88.9%), the sample was considered representative of the target population, and outcome measures were transparently reported, with attrition rates staying below 20%. However, only two studies (22.2%) implemented blinded assessment of outcomes, and just one study (11.1%) applied more than one outcome measure. A frequently observed limitation was the small sample size, as 5 studies (55.6%) included a relatively low number of participants.

## 4. Discussion

This systematic review synthesizes evidence from nine studies investigating VR-based multisensory interventions aimed at supporting cognitive improvement in individuals diagnosed with MCI. Results demonstrate consistent improvements in global cognitive function, with MoCA scores increased in 7/9 studies (78%), though executive function outcomes showed mixed results. One study reported significant improvement in depression as the psychological outcome, while feasibility assessments confirmed high patient tolerability and engagement. These findings underscore VR’s potential as a scalable cognitive rehabilitation tool while highlighting the need for standardized intervention frameworks and larger RCTs to establish clinical efficacy. While VR based interventions have shown promise in reducing depressive symptoms, lifestyle strategies such as regular physical activity, a healthy diet, and sufficient sleep have consistently provided stronger and more reliable benefits for mood and overall well-being in this population [13,14,17,19]. A key finding was the consistent improvement in global cognitive function, as indicated by increased MoCA scores in most studies. Reported improvements in MoCA scores, which generally range from 1.5 to 3 points, appear to approach or surpass the estimated minimal clinically important difference (MCID) of approximately 1.6 to 2.0 points based on data from stroke survivors. These findings suggest that such changes may hold meaningful implications for individuals’ everyday cognitive functioning [32]. This progress is consistent with the concept of neuroplasticity, that is, the brain’s ability to adjust and reorganize its neural connections in response to new experiences or inputs [9]. Recent studies indicate that the cognitive and emotional benefits of VR interventions for individuals with MCI may be driven by neuroplastic changes [5,9,10]. These changes are believed to result from the use of rich multisensory environments, novel tasks, and real-time feedback elements that actively engage brain regions such as the hippocampus and prefrontal cortex. Findings from neuroimaging and clinical research also suggest that the effectiveness of these interventions is influenced by factors like participant engagement, how often the training occurs, and how well the VR program can be tailored to individual needs. This underscores the value of designing VR experiences that are personalized and user focused [20,33,34,35]. Design elements also play a role in shaping user experience. For example, one study showed that spatial features such as urban furniture can encourage inclusive and interactive behaviors. This finding underscores the broader idea that environmental design influences human behavior, which aligns with current efforts to improve user engagement in virtual therapeutic settings [36]. Prior work in built environments has shown that spatial layout influences spatial cognition and wayfinding, supporting the value of structured design in both physical and virtual settings [37,38].

Additionally, VR training offers scalability, improving cognitive performance through the integration of visual, auditory, and sometimes haptic cues [9,11]. The studies used various tools for assessing global cognitive function, such as CERAD-K and MMSE, highlighting the need for standardized assessment tools (see Section 3.2). While improvements were observed in global cognition, the outcomes for executive function were mixed (see Section 3.3). This variability may be attributed to the multifaceted nature of executive function and the differing assessment tools, and VR task demands across studies. There was potential of VR for early dementia screening but also highlighted the need for careful selection of tools based on what cognitive domain is being tested [9]. Intervention acceptability and cultural differences are also important considerations. The studies included in this review were conducted in various regions, which may have influenced participants’ familiarity with technology, their preferences for how interventions were delivered, and their comfort in reporting psychological symptoms. While most studies reported high adherence rates and few adverse events, suggesting that the interventions were generally well accepted, there was limited information on users’ qualitative experiences and how the interventions were adapted to different cultural contexts. These aspects deserve more attention in future research.

Although wayfinding was not directly assessed in the included studies, several interventions involved tasks indirectly related to navigational abilities. Improvements in executive function and dual-task gait performance were reported using TMT-B, which engages cognitive flexibility and sequencing [21]. A VSEE simulation was used to mimic real-world spatial tasks, likely involving orientation and spatial memory [22]. Additionally, virtual cognitive–motor training showed gains in planning and working memory through TMT and digit span tasks [28]. Studies indicate that as many as 80% of individuals with MCI or early-stage AD exhibit pronounced difficulties with spatial navigation [5,6]. This cognitive decline significantly affects a person’s independence, safety, and general well-being, as it raises the likelihood of becoming disoriented or experiencing falls [7], and social isolation [8]. Difficulties in spatial navigation could act as an early, yet frequently unnoticed, indicator of preclinical AD [5]. Fortunately, VR interventions present a promising option for rehabilitation, utilizing repetitive, adaptive, and task-specific exercises to enhance wayfinding skills, spatial cognition, and overall navigation abilities in this population [10,21]. Beyond cognitive benefits, the reviewed studies suggest that VR interventions can positively impact psychological well-being, highlighting the potential of VR to address the emotional challenges associated with MCI and early AD [20,39]. These findings align with increasing research in this area indicating that engaging and stimulating activities can improve mood and reduce anxiety in older adults. Research has also shown that VR interventions can lead to enhancements in cognitive abilities, mood, and the ability to carry out daily tasks [40,41]. Since only one of the included studies reported a decrease in depressive symptoms, it is possible that individual biological characteristics such as genetic differences may influence how people respond to interventions. For example, the PTGS2 8473T>C gene variant has been significantly associated with depression in individuals with migraines. In addition, increased levels of inflammatory markers like IL-1β, IL-12, TNF-α, CRP, low CSF and serum BDNF, and altered adipokines are observed in individuals exhibiting both cognitive decline and depressive symptoms [42,43]. These results suggest that accounting for biological differences could improve the evaluation and personalization of VR interventions for cognitive and emotional support in individuals with mild cognitive impairment. Furthermore, feasibility assessments consistently demonstrated high patient tolerability and engagement. Participants generally reported enjoying the VR experiences and perceived both physical and cognitive benefits, suggesting strong adherence potential [39,44]. To ensure safety and minimize potential adverse effects, studies frequently employed SSQ to monitor cyber sickness symptoms. Notably, one study observed that the VR program not only enhanced cognition but also led to a reduction in discomfort across all SSQ items, suggesting that well-designed VR experiences can be both effective and comfortable for individuals with MCI [45,46]. These findings add to the expanding research highlighting the comprehensive benefits of VR interventions for people with MCI and early-stage AD. This systematic review provides a focused synthesis of cognitive training based on VR, specifically in individuals with MCI, drawing on both RCTs and single-group pre-post intervention studies to offer a comprehensive yet methodologically rigorous overview of current evidence. A key strength lies in the deliberate exclusion of observational, qualitative, and non-intervention studies, ensuring that all included research assessed measurable change attributable to a VR intervention. Unlike previous reviews that either limited inclusion to RCTs [24] or incorporated highly heterogeneous study types [23], this review strikes a balance by including intervention-based studies with pre-post outcome data, thereby improving internal validity without overlooking valuable smaller-scale trials [24,40]. Moreover, the review captures a broad range of outcomes, including cognitive function, executive function, psychological status, and functional ability that provides a more holistic understanding of VR’s therapeutic potential. Several limitations should also be acknowledged. A key limitation of this review is the high level of variation across studies, which makes it difficult to interpret and generalize the findings. The VR interventions differed in format, from immersive cognitive training to virtual supermarket tasks, as did the frequency, duration, and overall length of the programs. Studies used a range of outcome measures, including MoCA, MMSE, CERAD-K for global cognition, and various tools for executive and psychological functions, such as EXIT 25, TMT A and B, SCWT, GDS, MOSES, and QoL AD. Differences in participant age, education, MCI severity, and study design also added to the inconsistency. These factors likely affected the extent and type of cognitive gains reported and may explain the variability in outcomes across studies.

Most of the included studies involved small sample sizes and did not incorporate long-term follow-up, limiting the generalizability and long-term applicability of the observed outcomes [12,30]. Outcome assessor blinding was infrequently reported, increasing the potential risk of bias. We acknowledge that including non-randomized, single group pre post studies increases the risk of bias due to limited control over confounding factors. Because of the variability in interventions and outcome measures, a quantitative synthesis was not feasible. While meta-analysis is standard in our reviews, this narrative approach calls for cautious interpretation, as lower quality studies may overstate the benefits of VR interventions [47,48]. Although early-stage AD was within the scope of this review, no studies involving AD participants met the main inclusion criteria due to differences in design or outcome reporting. In addition, despite the inclusion of wayfinding and navigation accuracy as key outcomes of interest, no included studies directly assessed these domains. Future research should address these gaps by conducting large-scale, well-controlled trials that include early-stage AD participants and incorporate spatial navigation and real-world functional outcomes using standardized tools and longer-term follow-up. Such efforts would significantly enhance the ecological validity and clinical relevance of VR-based cognitive interventions.

## 5. Conclusions

This systematic review highlights promising evidence that VR-based multisensory interventions may support cognitive function and emotional well-being in individuals with MCI. While many studies demonstrated positive effects on overall cognition, outcomes related to executive function, daily functioning, and psychological well-being were less consistent, pointing to the need for more standardized intervention protocols. Notably, although early-stage AD was part of the inclusion criteria, no qualifying studies were identified for this group. Additionally, none of the included research directly evaluated spatial navigation, a significant gap considering its relevance in MCI. These results suggest that VR could offer meaningful, non-pharmacological benefits for cognitive rehabilitation; however, its broader clinical application is yet to be fully established. To strengthen the evidence base, future studies should focus on larger randomized controlled trials, consistent cognitive assessment methods, and extended follow-up periods to inform clinical practice and effectively integrate VR into neurocognitive rehabilitation strategies.

## Figures and Tables

**Table 1:** The risk of bias for the included studies was assessed using the NHLBI tool for non-randomized before-and-after clinical trials.

ID	Study	Q1	Q2	Q3	Q4	Q5	Q6	Q7	Q8	Q9	Q10	Q11	Q12	Quality Rating
29	Seri Maeng, 2021 [12]	Yes	Yes	Yes	No	Yes	Yes	Yes	No	Yes	Yes	No	NA	Good
103	Ying-Yi LIALO, 2020 [27]	Yes	Yes	Yes	No	No	Yes	Yes	Yes	Yes	Yes	No	NA	Fair
196	Jorge Buele, 2024 [20]	Yes	Yes	Yes	No	No	Yes	No	No	Yes	Yes	No	NA	Fair
239	Marta Mondellini, 2022 [22]	Yes	Yes	Yes	Yes	No	Yes	Yes	No	Yes	Yes	No	NA	Fair
311	Ji-Su Park, 2020 [28]	Yes	Yes	Yes	No	Yes	Yes	Yes	No	Yes	Yes	No	NA	Fair
327	Ying-Yi Liao, 2019 [21]	Yes	Yes	Yes	No	Yes	Yes	Yes	Yes	Yes	Yes	No	NA	Good
727	Wing Keung Ip, 2025 [29]	Yes	Yes	Yes	Yes	No	Yes	Yes	No	Yes	Yes	Yes	NA	Good
858	Lisa Sheehy, 2021 [30]	Yes	Yes	No	Yes	No	Yes	Yes	No	Yes	Yes	No	NA	Fair
946	Maho Tominari, 2021 [31]	Yes	Yes	Yes	Yes	Yes	Yes	Yes	No	Yes	Yes	No	NA	Good

**Table 2:** An overview of the primary characteristics of the included studies.

ID	Author, Country, Year	Sample Size	Age (Mean ± SD)	Sex (F/M)	Intervention Assessment	Type of Intervention	Outcome (Measurement)	Main Results
29	Maeng, Korea, 2021 [12]	31 MCI	73.2 ± 7.3	23/8	4 Weeks (8 sessions)	Cognitive training delivered through immersive virtual reality (VRCT)	Cognitive Function (CERAD-K)	The VRCT program demonstrated positive effects on cognitive performance and reduced symptoms of simulator sickness across all SSQ items in participants with MCI. Improvements were particularly noted in tasks such as word list recognition, word list recall, and Trail Making Test A (TMT-A) performance. However, no meaningful changes were detected in quality of life. (KQOL-AD) or depression levels (GDS) following the intervention.
							Depression (GDS)	
							Quality of Life (KQOL-AD)	
							Simulator Sickness Questionnaire (SSQ)	
103	Liao, Taiwan, 2020 [27]	18 MCI	75.5 ± 5.2	11/7	12 weeks (36 sessions)	Integrated physical and cognitive training using virtual reality (VRCT)	Daily functioning (Instrumental activities of daily living (IADL))	Participants in the VR group exhibited notable gains in overall cognitive function, delayed verbal memory, and instrumental activities of daily living (IADL) following the intervention.
							Brain activation (NIRS device)	
							Cognitive function (global cognition (MoCA), executive function (EXIT-25) and verbal memory (CVVLT))	
196	Buele, Ecuador, 2024 [20]	17 MCI	75.4 ± 5.7	10/7	6 weeks (12 sessions)	Virtual Reality	Cognitive function (MoCA-S)	The results showed significant improvements in cognitive function and geriatric depression within the group, with large effect sizes observed. However, no significant changes were noted in activities of daily living (ADL) performance, as anticipated.
							Depression (SGDS-S)	
							Functional ability (IADL-S)	
239	Mondellini, Estonia, 2022 [22]	15 MCI	75.7 ± 6.3	14/1	NA	Virtual Supermarket (VSEE-modified version)	Cognitive function (MoCA)	The MoCA scores varied between 21 and 25, with an average of 22.93 ± 1.44. For SSQ scores, no significant differences were found between pre- and post-intervention values, either in the total score or across the subscales.
							Simulator Sickness Questionnaire (SSQ)	
311	Park, Korea, 2020 [28]	18 MCI	75.8 ± 8.5	8/10	6 weeks (30 sessions)	Virtual reality-based cognitive–motor rehabilitation (VRCMR)	Trail Making Test A and B (TMT-A/B)	Within-group comparisons (pre- and post-training) reached statistical significance improvements in the MoCA, TMT-A, TMT-B, DST-forward, and DST-backward results for the VRCMR group (all *p* < 0.001).
							Cognitive function (MoCA)	
							Numeric rating self-report scale (NRSS)	
							Digit Span Test forward and backward (DST-forward/backward)	
327	Liao, Taiwan, 2019 [21]	18 MCI	75.5 ± 5.2	11/7	12 weeks (36 sessions)	VR-based TC (VRTC)	Executive function [Stroop Color and Word Test (SCWT) and trail making test (TMT) A and B]	The VR group demonstrated significant improvements in SCWT, single-task, motor dual-task gait performance, TMT-B, cognitive dual-task gait performance, and cadence DTC.
							Gait performance (gait speed, stride length, and cadence)	
							Dual-task costs (DTCs)	
							Walking tasks included single-task walking, walking while doing serial subtraction (cognitive dual-task), and walking while carrying a tray (motor dual-task).	
727	Ip, China, 2025 [29]	9 MCI	73.6 ± 6.1	9/0	8 weeks (16 sessions)	VirCube VR	Cognitive function (HK-MoCA)	The results indicated that the HK-MoCA test showed significant improvement following the VR intervention. However, no evidence was found to support changes in executive functions within this group.
							Participants’ executive functions such as their ability to retain information, switch between tasks, and process information efficiently by using the Trail Making Tests (TMT-A and TMT-B).	
858	Sheehy, Canada, 2021 [30]	11 MCI	78 ± 7	4/7	6 weeks (30 sessions)	Home-based VR exercise	Practicality and safety of using VR in a home setting	Participants completed 99% of the assigned exercises, and no significant adverse events occurred. While most participants enjoyed the VR program and reported physical benefits, fewer noted cognitive improvements. After 6 weeks, no changes were observed in physical or cognitive outcome measures.
							Early findings across various physical and cognitive clinical outcomes.	
946	Tominari, Japan, 2021 [31]	26 MCI	85.1 (69–98)	19/7	Follow up: 8 weeks	Virtual Reality	Cognitive function (MMSE)	Cognitive function, measured using the MMSE, showed improvements after the intervention with VR panoramas, suggesting that reminiscence therapy enhanced cognitive abilities. The average total score on the revised PGC Morale Scale rose after the intervention. However, no significant changes were found in the pre- and post-intervention scores on the Trail Making Test and Word Fluency Test, according to the MOSES scale.
							Revised PGC morale scale	
							Multidimensional observation scale for elderly subjects (MOSES)	
							Trail making test parts A and B (TMT-A, TMT-B)	
							Word fluency test (WFT)	

## Data Availability

The derived data supporting the findings of this study are available from the corresponding author upon reasonable request.

## Abbreviations

The following abbreviations are used in this manuscript.

MCI	Mild Cognitive Impairment
AD	Alzheimer’s Disease
VR	Virtual Reality
PRISMA	Preferred Reporting Items for Systematic Reviews and Meta-Analyses
IADL	Instrumental Activities of Daily Living
SSQ	Simulator Sickness Questionnaire
NHLBI	National Heart, Lung, and Blood Institute
VRCT	VR-based Cognitive Training
VRCMR	VR-based Cognitive–Motor Rehabilitation
MMSE	Mini-Mental State Examination
MoCA	Montreal Cognitive Assessment
CERAD-K	Consortium to Establish a Registry for Alzheimer’s Disease-Korean Version
GDS	Geriatric Depression Scale
MOSES	Multidimensional Observation Scale for Elderly Subjects
QoL-AD	Quality of Life–Alzheimer’s Disease
